# Exploratory laparotomy and drainage of a left pelvic abscess: a case report

**DOI:** 10.1093/jscr/rjag613

**Published:** 2026-07-21

**Authors:** Frederick M Tiesenga, Chetachukwu Eze, Jinwook Kang, Osman Kayani, Osadiame Uduehi

**Affiliations:** General Surgery, Suburban Surgery Center, 1950 N. Harlem Ave, Elmwood Park, IL 60707, United States; Medicine, St. George’s University School of Medicine, University Center, St. George, Grenada, West Indies; Medicine, St. George’s University School of Medicine, University Center, St. George, Grenada, West Indies; Medicine, Windsor University School of Medicine, Island Main Rd, Cayon, Saint Kitts and Nevis, West Indies; Medicine, Washington University of Health and Science School of Medicine, Sea Star Drive, San Pedro, Belize, West Indies

**Keywords:** gastrointestinal surgery, intra-abdominal abscess, sepsis, exploratory laparotomy, immunosuppression

## Abstract

Intra-abdominal abscesses within the peritoneal cavity and pelvis are formed in response to infection or inflammation, often following trauma, surgery, or chronic inflammation. Immunocompromised patients are at high risk of developing postoperative abscesses. Affected patients may present atypically or with signs of sepsis, even without abscess rupture. In this case, a 58-year-old male with stage III chronic kidney disease secondary to sarcoidosis & prostate cancer presented to the emergency department with 10 days of back and left hip pain, lower abdominal pain, and early signs of sepsis. Following an urgent care visit for his symptoms 2 days prior, his condition deteriorated, and imaging showed a left pelvic mass. This prompted emergency exploratory laparotomy, revealing a left pelvic sidewall abscess with abacterial purulent fluid without bowel perforation. We highlight the management of this patient’s abscess and his post-operative care, alongside the contributing factors that led to his abscess formation.

## Introduction

Intra-abdominal abscesses (IAA) are collections of walled-off pus arising from infectious or inflammatory processes, localized adjacent to the terminal ileum, within the peritoneal cavity, retroperitoneum, or intra-mesenteric regions [1, 2]. Causes include infections, post-surgical complications, trauma, and chronic inflammation, contributed by diaphragmatic contraction, peritoneal fluid lymphatic filtration, and sequestration of hard-to-eliminate foreign substances. Their formation follows a complex physiological process involving microbial invasion and immune response, leading to localized contamination within the abdomen.

Source control is fundamental to treating IAAs. They generally develop as the host’s response to peritoneal inflammation secondary to an underlying infection, usually enteric bacteria. *Escherichia coli* and *Bacteroides fragilis* are the most frequently isolated pathogens [3]. Aseptic abscesses are uncommon, associated with foreign bodies, chemical irritants, or autoimmune conditions [4].

Abscess formation risk rupture, leading to sepsis. However, patients may present with early signs of sepsis prior to rupture, typically fever and tachycardia due to the abscess wall producing proinflammatory cytokines [tumor necrosis factor alpha (TNF-α), interleukin 1-beta (IL1-β), interleukin 6 (IL-6)]. These cytokines enter local vessels, predominately the portal venous system, and eventually systemic circulation. Less frequently, bacteria can translocate from the abscess directly to the bloodstream, thus, clinicians should always evaluate bacteremia for source control in the presence of sepsis.

## Case

A 58-year-old male presents with 10-days of back and left hip pain & 3-days of diffuse lower abdominal pain with constipation. He visited an urgent care clinic 2-days earlier, but he since developed worsening abdominal pain, a fever, and chills. He has stage III chronic kidney disease (CKD) secondary to sarcoidosis, managed with Azathioprine, and prostate cancer. He underwent a prostatectomy 6 months earlier and an appendectomy when young. His vitals highlighted fever (100.4°F/38°C), tachycardia (100 bpm), and hypotension (94/50 mmHg).

**Figure 1:**
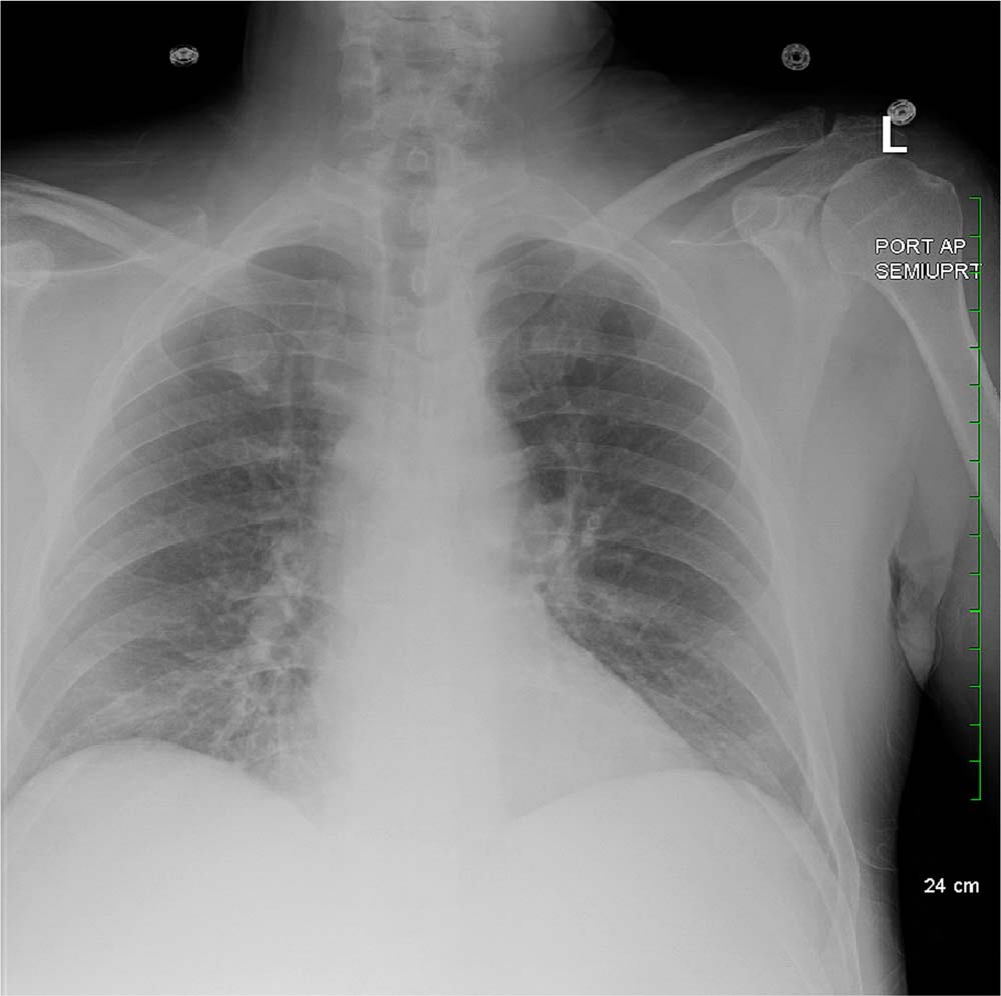
Pre-operative chest X-ray (anterior–posterior).

**Figure 2:**
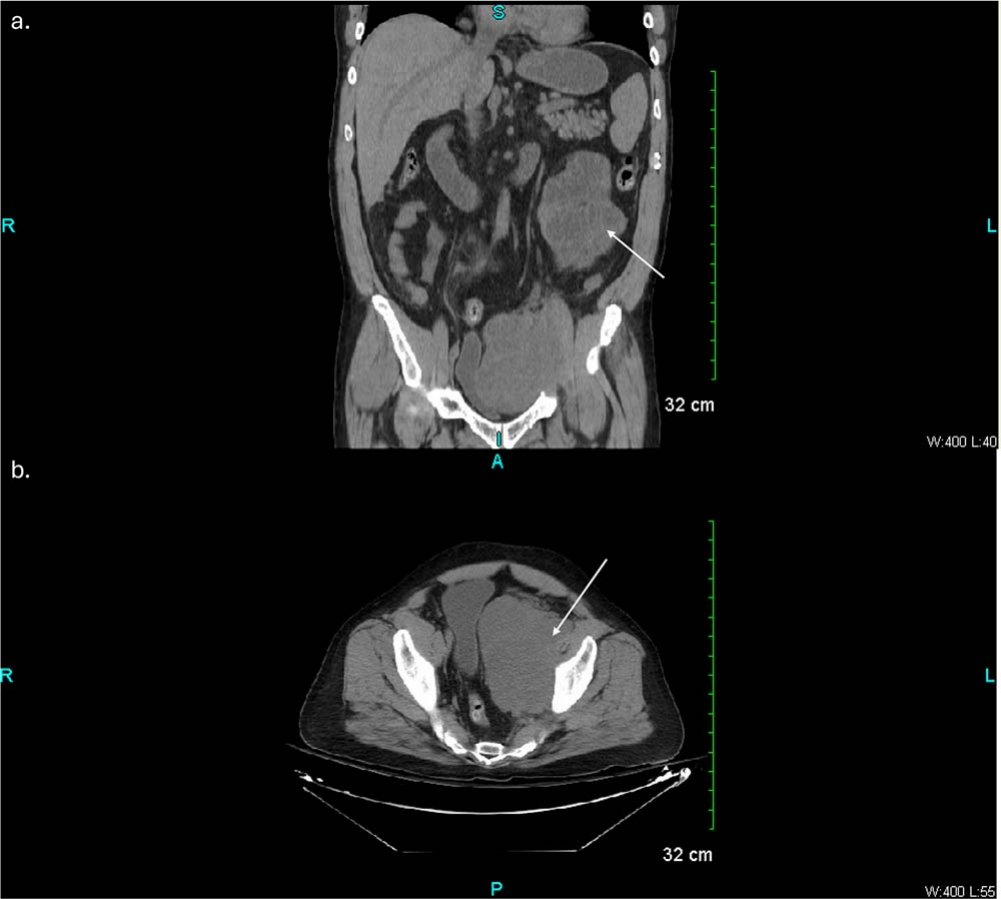
Pre-operative abdominal/pelvic CT scan without contrast (coronal and axial views).

Physical examination noted a non-distended abdomen with diffuse tenderness, rebound, and guarding. Laboratory analysis revealed anemia (hemoglobin = 9.5 g/dl; hematocrit = 26.3%), leukopenia (leukocyte count = 3 × 10^3^ mm^3^), elevated blood urea nitrogen (147 mg/dl) and creatinine (5.48 mg/dl), and metabolic acidosis (anion gap = 27). A chest x-ray revealed no cardiopulmonary culprits (Fig. 1), but an abdominal/pelvic computed tomography (CT) scan without intravenous (IV) contrast showed a pelvic soft tissue mass (12.4 × 10.0 × 11.3 cm) encasing the left psoas major and iliopsoas muscles near the sigmoid colon and left kidney, displacing the bladder rightward (Fig. 2). The scan raised suspicions of reactive ileus or enteritis due to the appearance of multiple distended small bowel loops and mild mesenteric edema, raising concerns of a perforated bowel and acute abdomen requiring urgent surgical intervention.

**Figure 3:**
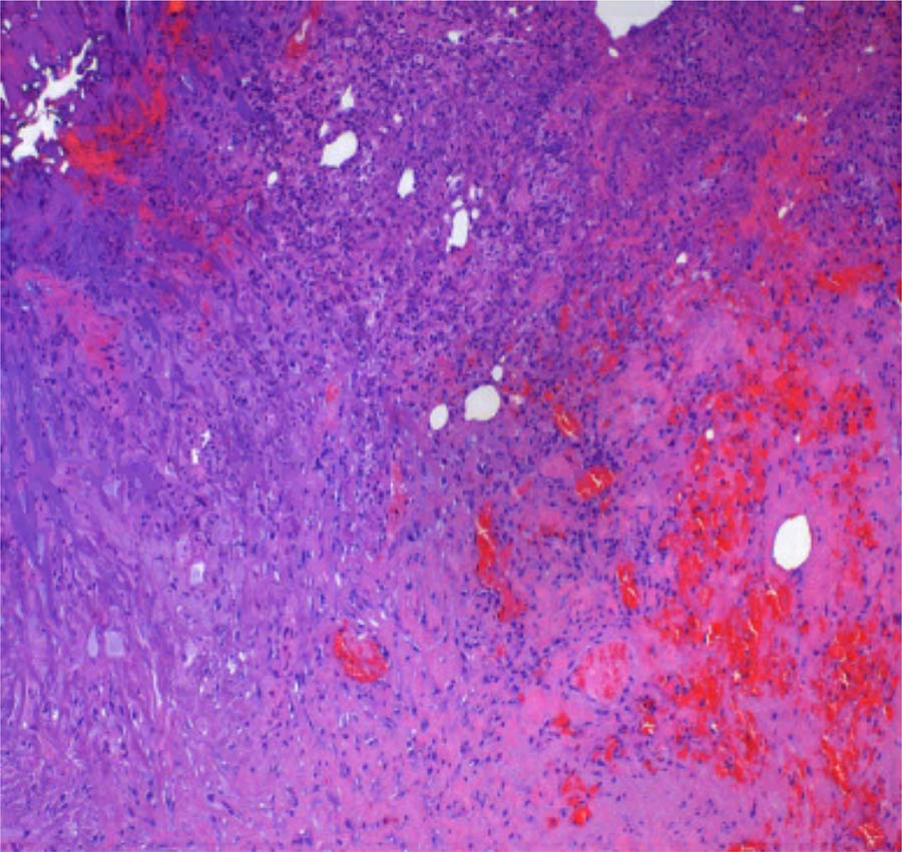
H&E-stained section of the biopsied pelvic abscess cavity.

Copious purulent green fluid was encountered within the peritoneum. Abdominal exploration unveiled appendectomy adhesions, fibrinous rings, no perforations, and an abscess on the left pelvic sidewall. Pelvic abscesses are typically drained percutaneously with ultrasound assistance, as open drainage risks infection and postoperative bleeding [5], but this abscess was novel, requiring immediate drainage. One liter of fluid was drained and tested culture-negative, and the abscess cavity was sectioned for pathological analysis, revealing fibroadipose tissue negative for prostatic malignancy (Fig. 3). Blood cultures grew Group C *Streptococcus* (GCS). The abdominal cavity was irrigated afterwards, and two Jackson-Pratt drains were inserted before suturing.

The procedure was well-tolerated, and prophylactic Piperacillin/Tazobactam, and later Amoxicillin/Clavulanate and Ciprofloxacin, was administered. Vasopressors were also given for hypotension management. He spent the first 3 days post-operation in intensive care and was discharged by the ninth day.

## Discussion

Our patient’s abscess likely originated hematogenously or postoperatively, supported by his GCS bacteremia and recent prostatectomy. Abscesses are rare complications of laparoscopic prostatectomy, occurring in 1.1% of cases [6], so it’s reasonable to examine potential causal factors. However, there is uncertainty regarding the extent and influence of these or other factors in this case, and we aim to hypothesize what likely occurred based on the patient’s history & presentation.

The patient’s blood count showed leukopenia—an adverse effect of Azathioprine, requiring frequent monitoring to avoid pancytopenia [7]. Azathioprine is preferred to the more conventional Methotrexate in those with advanced CKD like this patient, as it can worsen kidney injury [8]. With leukopenia comes immunosuppression, which may have led to the patient’s GCS bacteremia. GCS is found naturally in the oral cavity, oropharynx, and nasopharynx, but it can enter the bloodstream in patients with diabetes, immunosuppression, malignancy, or IV drug use [9].

The patient’s bacteremia may have seeded to his left iliopsoas muscle, causing pyomyositis and abscess formation. Pyomyositis typically occurs in extremities and is tied to IV drug use [10]. Given the patient never utilized IV drugs and has a pelvic abscess, his case is remarkably rare. The iliopsoas muscle is close enough to the prostate that it may have been disrupted during the patient’s prostatectomy, leaving it vulnerable to the body’s GCS. The patient’s Azathioprine usage may have delayed recovery post-prostatectomy due to immunosuppression, increasing the risk of pyomyositis and abscesses.

The 2017 World Society of Emergency Surgery guidelines for managing intra-abdominal infections recommends CT-guided percutaneous drainage of IAAs for its minimal invasiveness and low infection risk [11]. However, this abscess wasn’t definitively identified on imaging, and the patient lacked leukocytosis, which usually presents with pain and fever [12]. Given the iliopsoas muscle’s proximity to surrounding abdominal structures, abscesses there can yield overlapping symptoms, causing misdiagnosis or delayed recognition. This patient showed signs of perforation and required immediate response, necessitating exploratory laparotomy [13], making it the preferred first-line treatment in immunocompromised patients with worsening features. The involvement of GCS necessitates broad-spectrum empiric antibiotics and close monitoring for dissemination or abscess recurrence [11]. It may be worthwhile to re-examine and update guidelines on the management of similarly immunocompromised patients undergoing intra-abdominal procedures to minimize the risk of post-operative pyomyositis and abscesses.

## Conclusion

This case highlights the need for early IAA detection, especially in patients exhibiting vague or misleading symptoms. Physicians should recognize and address at-risk individuals with a high degree of clinical suspicion since IAAs may exhibit atypical symptoms requiring exploratory laparotomy instead of ultrasound-guided percutaneous drainage. Given the rarity of this patient’s case, it is reasonable to re-examine and update current guidelines for the post-operative care of immunocompromised patients to reduce their risk of developing IAAs.
